# An effective and concise device for detecting cold allodynia in mice

**DOI:** 10.1038/s41598-018-31741-7

**Published:** 2018-09-18

**Authors:** Yonglan Ruan, Leying Gu, Jinjin Yan, Jun Guo, Xiao Geng, Hao Shi, Guang Yu, Chan Zhu, Yan Yang, Yuan Zhou, Changming Wang, Zongxiang Tang

**Affiliations:** 1 School of Medicine and Life Sciences, Nanjing University of Chinese Medicine, 138 Xianlin Rd, Nanjing, 210023 Jiangsu China; 2 Key Laboratory of Chinese Medicine for Prevention and Treatment of neurological diseases, Nanjing University of Chinese Medicine, 138 Xianlin Rd, Nanjing, 210023 Jiangsu China; 3 State Key Laboratory Cultivation Base for TCM Quality and Efficacy, Nanjing University of Chinese Medicine, Nanjing, 210023 China; 4 Key Laboratory of Drug Target and Drug for Degenerative Disease of Jiangsu Province, Nanjing University of Chinese Medicine, Nanjing, 210023 China

## Abstract

Detection of cold allodynia is a very important aspect in the study of pain behavior. An effective and concise device for detecting cold pain has always been the hope of many researchers. Here, an easily produced and operated cold plate device is presented for the assessment of cold allodynia in mice. The device used to detect cold allodynia has two components: a chamber consists of a cylinder for animal experiment and a cube box around the chamber for holding ice to keep temperature stable. In the testing chamber, a mouse was placed on the circular plexiglass plate steady at 4 °C above ice for five minutes. The tested mouse will lift its paw when exposed to the cold plate. The number of lifts will present animal’s response to the degree of cold stimulation. To evaluate this approach, three commonly used pain models of mice were tested: formalin test, bone cancer pain (BCP), and chronic constriction injury (CCI). As is reported in other literatures, these three pain mice models showed increased sensitivity to cold stimulation. The new device is indeed suitable for detecting cold allodynia behavior in mice. Comparisons with existing devices of detecting cold allodynia, such as the cold plate in the market (UGO, Panlab, Columbus, etc.), the new device has the advantages of low cost, simple operation and easy popularization and can detect cold allodynia behavior of mice very well. This is a very practical and economical device to detect cold allodynia behavior.

## Introduction

Most animal models of acute and chronic pain rely on reflective assays for evaluation of nociception, such as mechanical stimulation, cold stimulation, heat stimulation, and chemical stimulation^1,2^. Of all these methods, the detection of cold stimulation is the most difficult to operate because a stable, low temperature is necessary. The acetone test has been adopted by researchers for detecting cold allodynia^3–5^. A drop of acetone applied to the mid-plantar area of the hind paw and the resulting behavior monitored for one minute. The temperature drops to 17 °C from room temperature. The scores obtained by arbitrary judgment. Moreover, there was another objective method of cold plated testing for detecting cold allodynia. Cold plate testing was commonly used by placing the mouse on the cold plate and counting the frequency of withdraw responses^6–8^. However, the expensive price of the cold plate limited the use of researchers. Considering the arbitrary judgment of the acetone test and the cost of the UGO cold plate, we present a new device that can be easily made and operated: a plexiglass device (temperature of the plate is steady at 4 °C). Then the withdraw responses could be judged objectively. The new device not only provides a good way for detection of cold allodynia, but also be easily popularized.

## Results

### A new device for detecting cold allodynia

**Figure 1:**
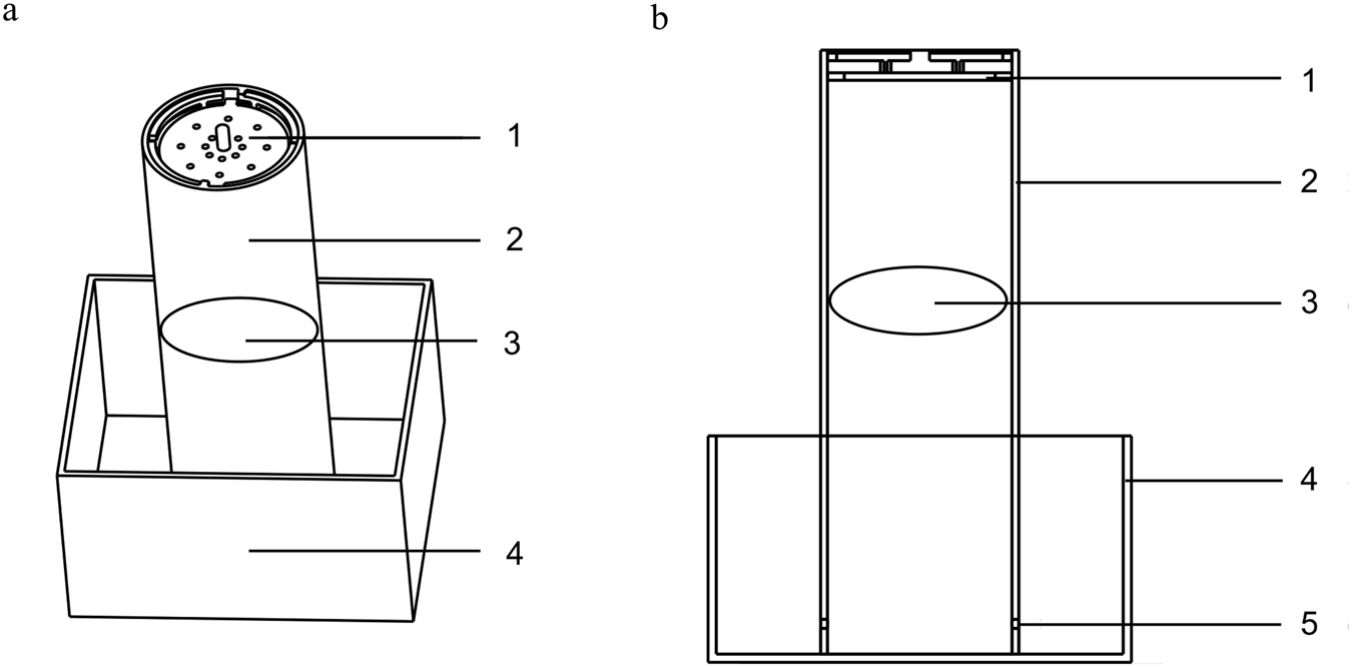
The new device model diagram for detection of cold allodynia. (**a**) The device consists of 4 parts: (1) the cover to prevent the mouse from jumping out, (2) the restricting cylindrical testing chamber, (3) the circular cold plate, and (4) the plexiglass cubic box. The holes in the lid are for the mouse to breathe. (**b**) The side view of the new device. The holes (5) on the side of the lower part of cylindrical testing chamber are for the flow of melted ice water.

**Figure 2:**
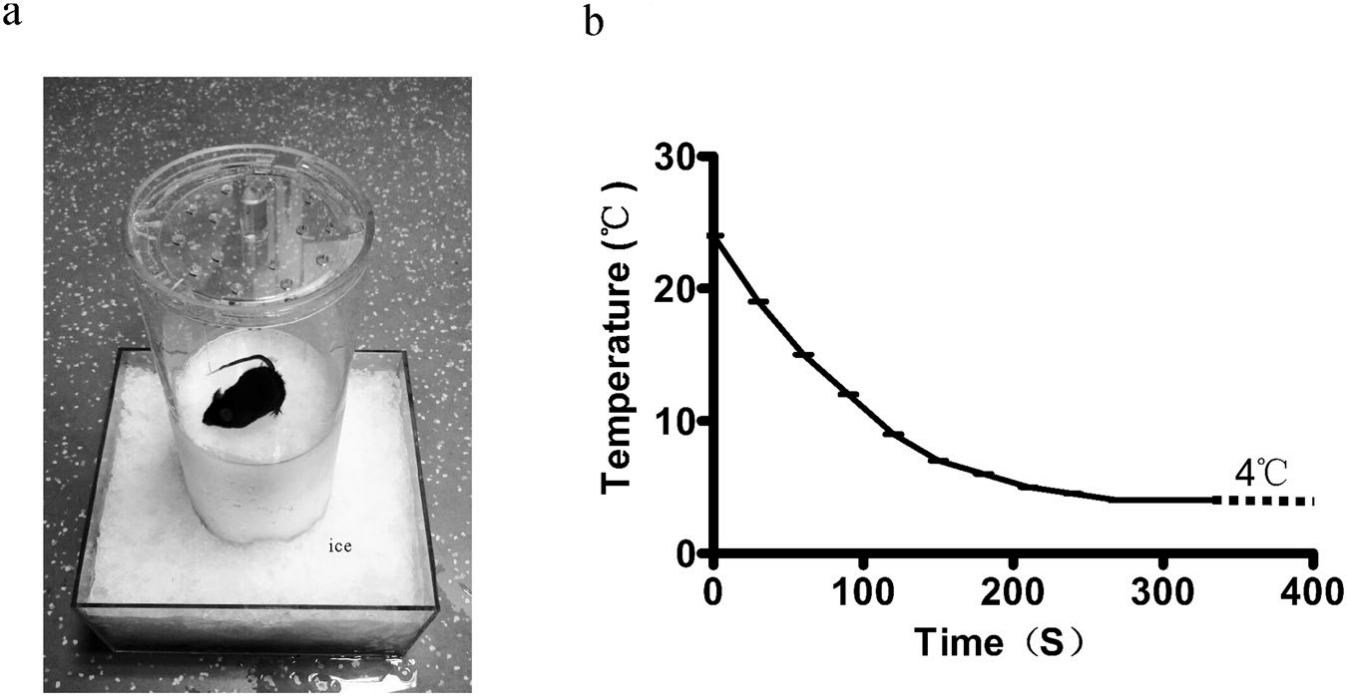
The detection of cold pain behaviors and the temperature variation curve of the plate on the ice. (**a**) A pattern for animal behavior detection. The lower half of the cylindrical testing chamber and the square container outside were filled with ice. The tested mouse was placed on the cold plate. (**b**) The relation curve of temperature and time variation. When the circular plate is tightly pressed against the ice, the temperature of the plate surface varies with time and can be stabilized at 4 °C in 5 minutes.

The newly generated device consists of three parts: the lid, the restricting cylindrical testing chamber and the plexiglass cube box (Fig. 1a,b). There are some holes in the lid for the mouse to breathe and the lid can prevent the mouse from jumping out. The restricting cylindrical testing chamber is 405 mm long, the chamber is 122 mm in diameter. The cube box was 250 × 280 × 150 (mm). Fresh ice is added into the chamber and the cube box. A circular plate (2 mm thick, 120 mm in diameter) made of plexiglass is placed on the ice (Fig. 2a). The temperature of the circular plate drops and stabilizes at 4 °C within five minutes after pressing the circular plate on the ice (Fig. 2b). It’s simple and easy for researchers to operate. The temperature of the cold plate (4 °C)-induced responses of the hind paw is suitable for evaluating cold allodynia of mice.

### Detection of cold allodynia in formalin-induced acute pain by the new device

**Figure 3:**
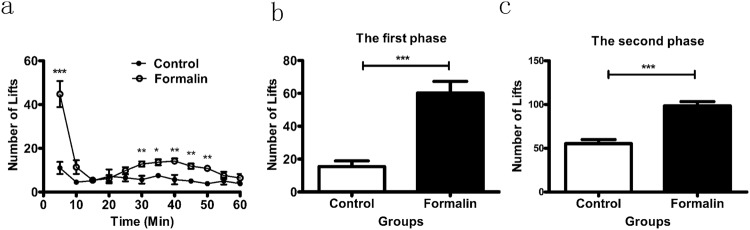
Detection of cold allodynia in formalin test. (**a**) The number of lifts was recorded for 60 min after the injection of 4% formalin into the right hindpaw plantar surface. Two phases of pain behavior were obviously different between the formalin group and the control group. (**b**) The first phase of the formalin test on the cold plate. (**c**) The second phase of the formalin test on the cold plate. N = 7, *p < 0.05, **p < 0.01, ***p < 0.001.

To detect the cold allodynia in acute pain, we employed the mouse formalin test, a model of chemically induced pain^9^. The lifts were recorded within 60 min after subcutaneous injection of 4% formalin into the right hind paw plant on the new cold plate. There were obviously two phases of cold allodynia in 60 min compared with the control group (Fig. 3a, n = 7). The lifts in both the first and second phases were obviously different with the control group (Fig. 3b,c). Compared to previous researches^9–13^, this new device can truly reflect the cold allodynia in mouse formalin test-induced acute pain.

### Detection of cold allodynia in bone cancer pain by the new device

**Figure 4:**
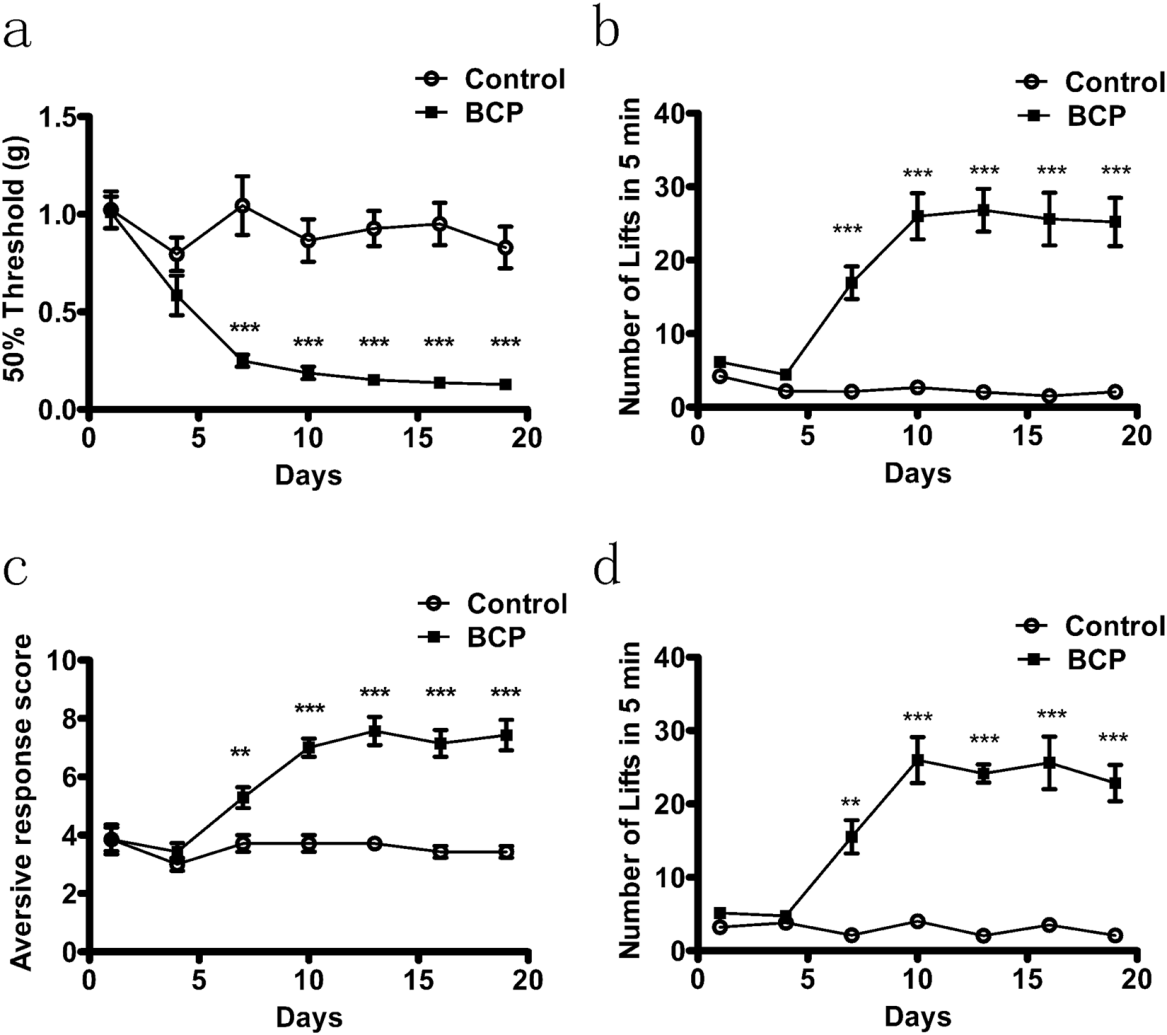
Cold allodynia behaviors observation of bone cancer pain in mice. (**a**) Von-Frey testing of BCP and the controlled comparison. The threshold of MWT varies with time. The 10^th^ day of bone cancer pain model showed significant differences. (**b**) Cold plate testing of the new device over 20 days after BCP and controlled comparison in mice. Values represent the changes from the baseline lifts of ipsilateral paws that were recorded on day 0 after operation. (**c**) Cold response in the ipsilateral paws of mice in acetone test over 20 days after BCP and controlled comparison. (**d**) Cold plate testing of UGO over 20 days after BCP and controlled comparison in mice. Values represent the changes from the baseline lifts of ipsilateral paws that were recorded on day 0 after operation. N = 7, **p < 0.01; ***p < 0.001.

To verify the detection of cold allodynia by the new device, a BCP operation and a sham operation were applied to mice. Then we recorded the mechanical withdrawal threshold (MWT) on the1^st^, 4^th^, 7^th^, 10^th^, 13^th^, 16^th^ and 19^th^ day after operation. The MWT values in the BCP groups obviously reduced compared to those in the sham operation groups, indicating that the BCP operation was successful (Fig. 4a).

Next, we detected the cold allodynia of bone cancer pain by our new device. The testing mouse was placed on the cold circular plate, the lifts of the hind paws were calculated within five minutes. There was difference of lifts compared to those in the sham operation groups (Fig. 4b). To confirm our device, the method of acetone test was applied. As we hypothesized, the results of the acetone test were similar to our new device (Fig. 4c).

To confirm our new device has the same function with cold plate of UGO to detect cold allodynia, cold plate of UGO was used to detect the cold pain. At last, the results of our new device are similar to cold plate (Fig. 4d, UGO Basile) in the market.

### Detection of cold allodynia in the CCI model by the new device

**Figure 5:**
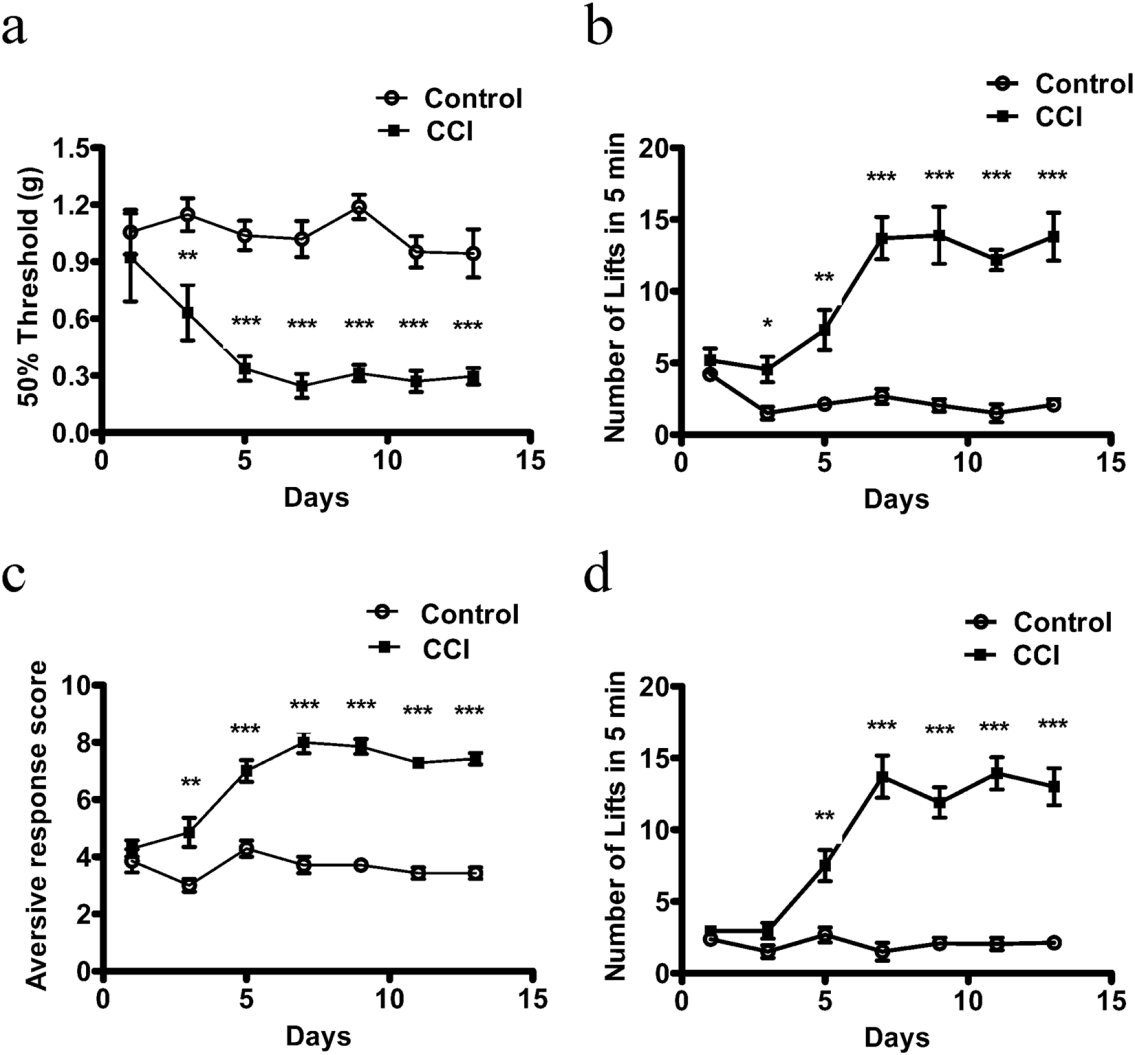
Detection of cold allodynia behaviors of chronic constriction injury of sciatic nerve in mice. (**a**) Von-Frey testing over 14 days after CCI or controlled comparison of mice. Values represent the changes from the baseline MWT values that were recorded on day 0 after operation. (**b**) Cold plate testing of the new device over 14 days after CCI and controlled comparison of mice. Values represent the changes from the baseline lifts of ipsilateral paws that were recorded on day 0 after operation. (**c**) The cold response in the ipsilateral paws of mice in acetone test was significantly higher than that of the control group at 14 days after CCI operation. (**d**) Cold plate testing of UGO over 14 days after CCI and controlled comparison of mice. Values represent the changes from the baseline lifts of ipsilateral paws that were recorded on day 0 after operation. N = 7, **p < 0.01; ***p < 0.001.

CCI is another mouse model of chronic pain that is adopted in the study of neuropathic pain. The CCI operation model was applied to detect the cold allodynia by our new device. We recorded MWT on the 1^st^, 3^rd^, 5^th^, 7^th^, 9^th^, 11^th^ and 13^th^ day after the CCI operation. The MWT values in the CCI groups obviously reduced compared with those in the sham operation groups, indicating that the CCI operation was successful (Fig. 5a). The cold allodynia of CCI was detected through the new device. The testing mouse was placed on the cold circular plate and the lifts of a hind paw were calculated within five minutes. The lifts of the operation groups were obviously more than sham group (Fig. 5b). To confirm the result, the acetone test method was applied. As we hypothesized, the results of the acetone test were similar to our new device (Fig. 5c). The new device of detecting cold allodia may represent an objective way to evaluate cold allodynia in chronic pain models in mice.

To confirm our new device has the same function with cold plate of UGO to detect cold allodynia, cold plate of UGO was used to detect the cold pain. At last, the results of our new device are similar to cold plate in the market (Fig. 5d, UGO).

## Discussion

The evaluation of cold allodynia is an important index and parameter for the study of pain behavior. We present an easily made device for detection of cold allodynia that objectively reflects cold pain. Presently, there are some other methods for detection of cold allodynia, such as the acetone test. In the acetone test, the temperature drops from room temperature to 17 °C after the drop of acetone, then the scores or paw lifts are evaluated^4,14^. However, the detection of some lower temperature receptors is powerless. In the cold plate testing, mouse was placed on a cold plate maintained at 4 °C, and the number of paw lifts was counted. The cold stimuli was also tested by a chilled (4 °C) brass probe, and the responses were evaluated^2^. All of these methods and devices were either subjective or complicated. The new device avoids the mentioned defects and can be easily popularized.

The new device was made of plexiglass and the temperature of the testing plate was steady at 4 °C (Fig. 2b), as the cold testing plate was placed on fresh ice. The plexiglass device readily conducts cold temperatures. Its transparent feature is also suitable for video recording. It’s easy to popularize because plexiglass is unfragile and cheap. However, there are still some shortcomings. It is not adapted for measuring orofacial pain for the limitation of the new device. In order to avoid a bias in hand quantitation, the tests should perform with an experimenter blind to the group.

To avoid the cold nociceptive pain, mice lift their hind paws occasionally. Therefore, the lifts of hind paw were counted for evaluation of cold allodynia. For getting an exact result, a standard assessment is necessary during the research. The behavior of lifting showed in the video (Video 1). From the video, our relatively objective method reflects the cold pain of mice.

In the formalin test, we divided the mice into two groups after we injected formalin to induce inflammatory pain, one group of animals at room temperature, another group of animals on the cold plate, then observed their behavioral responses within 60 minutes. A large number of literatures have shown that in the process of inflammatory pain the mice represented two-phase reactions including acute phase and tonic phase^13,15,16^. Acute phase lasts about 5 min resulted from direct chemical activation of myelinated and unmyelinated nociceptive afferent fibers. After a short quiescent period, tonic phase begins. This phase lasts approximately 40 min which is considered to be associated with central sensitization^17,18^. There are also two phase in cold pain behavior response in formalin test, probably because TRPA1 plays an important role in the model. TRPA1 has been shown to be associated with formalin induced pain^15,19^ which is a cold-sensitive ion channel^4,14,20^. So we tested the performance of cold pain in the inflammatory state.

Compared with the common cold pain detection equipment, the new device has the following advantages. First, the low cost is the biggest advantage. The price of a UGO cold plate in the Chinese market is about $ 14000, but the cost of making the new device is about $ 35. Second, the new device is easy to repair when it is necessary. Every part of the new device is easy to buy in the market and you can also make it by yourself. However, the repair of UGO cold plate is more complex and requires a longer period of time. Third, the detection effect of cold allodynia is similar between the new device and the cold plate of UGO. Therefore, this new device can meet the requirements of our experiment such as detecting cold allodynia of neuropathic pain, bone cancer pain and formalin induced pain.

## Materials and Methods

### Device and function

The device consists of a cylindrical testing chamber in a 250 × 280 × 150 (mm) plexiglass plate cube box (Fig. 1a,b) with a cover. The cube box and cylindrical testing chamber filled with fresh ice and compressed tightly. The thickness of ice in the chamber was about 250 mm. The cover fixed to the top of the chamber prevents the mouse from jumping out. The holes in the bottom of the chamber are for the flow of melted ice water. After fixing the ice, a 2 mm thick circular plexiglass plate placed tightly on the ice in the chamber (Fig. 2a). A surface temperature sensor (DT1311, Chengxin instrument, Shenzhen, China) was used to detect surface temperature of the plate. After putting the temperature sensor on surface of the plate, we could read the variation of temperature on the monitor. The temperature of plate was steady at 4 °C for five minutes after placing on the ice (Fig. 2b). For testing the cold allodynia, the mouse placed on the cold plate, the frequency and time of lifts calculated for evaluation of cold allodynia.

### Animals

This study was approved by the Animal Care and Use Committee of Nanjing University of Chinese Medicine (Nanjing, China). Experiments were conducted according to the animal research ethical guidelines of the International Association for the Study of Pain. Male C57BL/6 mice were housed in groups of four per cage in our animal center, with free access to food and water. Only healthy animals weighing 15–20 g and displaying normal water and food intake were included in the study. All behavioral tests were performed with an experimenter blind to the group.

### Formalin test

Mice were injected with 10 μl of 4% formalin into the right hind paw plantar surface, and were placed on the cold circular plexiglass plate in the round cylindrical restriction device. The frequency of lifts in 60 minutes were calculated for evaluation of cold allodynia. It has been reported that the 60 minutes test time course is necessary in the process of detecting the pain induced by formalin^9,19,21^.

### Bone cancer pain model

Lewis lung carcinoma (LLC; Shang Hai, China) cell line was cultured at 37 °C in DMEM medium supplemented with 10% fetal bovine serum in a humidified atmosphere containing 5% CO_2_ as previously reported^22–24^. After five days in culture, the cells were trypsinized, suspensions of 1 × 10^6^/ml tumor cells in PBS were prepared and ready for femur injection (10^6^). A mouse model of bone cancer pain was established by femur injection of LLC as previously described^22–24^. Mice were anaesthetized with isoflurane (Yuyan instrument, Shanghai, China) and restrained in a supine position. The right patellar surface of the femur was carefully exposed, and the middle of the patellar surface of the femur was punched by an electric drill. A 23-gauge needle was inserted in the bone marrow cavity through the hole. A volume of 10 μl LLC (1 × 10^6^) or vehicle (1 × PBS) was injected into the femur bone cavity by a 20 μl Hamilton syringe. After the injection, the site was cleaned by cotton balls and then sealed by bone wax. Finally, the wound was closed and sterilized. After the operation, the mice were positioned supine until they recovered from the anesthesia. All surgical procedures were performed under sterile conditions.

### Chronic constriction injury (CCI)

Mice were anaesthetized with isoflurane (Yuyan instrument, Shanghai, China) and restrained in a lateral position. A surgical incision was made at the thigh root midline, and the right sciatic nerve was exposed in the middle of the thigh. Three ligatures were tied around the nerve by using 4-0 silk braided cord (Shanghai Pudong Jinhuan Medical Products Co., Ltd) with 1 mm space^25^. The force of ligation was based on leg reflex. The length of the nerve affected was 4–5 mm. After the operation, the wounds were sutured shut and the animals were positioned supine and kept that position until they recovered from the anesthesia. A sham operation was performed in the same manner except for sciatic nerve ligation. All surgical procedures were performed under sterile conditions.

### Mechanical allodynia

Animals were acclimated to the testing environment for 30 minutes before the initiation of behavior tests. Animal behaviors were analyzed by investigator who was blind to the grouping. Mechanical allodynia was assessed by measuring the paw withdraw threshold with a set of Aesthesio Von Frey filaments (0.04–2 g). Mice were placed on an elevated metal grid (100 cm × 50 cm). The filament was applied to the plantar surface at a vertical angle for up to 3 s from the bottom. Fifty percent MWT values were determined using the up-down method^26^.

### Acetone test

Cold allodynia was evaluated by the acetone test as before^5,27^. Mice were placed on a plexiglass plate (100 cm × 50 cm). A drop (50 μl) of acetone was applied to the mid-plantar area of the hind paw, and the behavioral results were monitored for 1 minute. A score of 0 indicated no response; 1 quick withdrawal or flicking; 2 prolonged withdraw or repeated flicking of the paw; 3 repeated flicking of the paw with licking of the paw. Acetone was applied three times to each paw alternately and the responses were scored. Cumulative scores were then obtained by adding the three scores for each of the mice.

### Cold plate tests by Ugo Basile

Mice were placed on a cooled metal plate (Cold Plate, UGO Basile)^28^. The metal plate was cooled down to 4 °C and the number of reactions (hind paw lifts) during 5 minutes were counted.

### Data analysis

All data are presented as mean ± SEM. The number of animals used in each study was based on our experiences and similar studies. After the experiments were completed, no data point was excluded. The results of formalin test were analyzed by using Student’s t-tests (SPSS version 16.0) to compare the behavior results. Repeated-measures ANOVA (SPSS version 16.0) was used to compare the behavioral results of CCI and BCP model. *P* < 0.05 was considered statistically significant in all tests.

## Electronic supplementary material

Legend of video

Lifts
