# Supplementary material for: An effective and concise device for detecting cold allodynia in mice
Source: Sci Rep. 2018 Sep 18;8:14002. doi: 10.1038/s41598-018-31741-7 (PMC6143538; doi:10.1038/s41598-018-31741-7)
Supplement: Supplementary file 1 — Legend of video [file 41598_2018_31741_MOESM1_ESM.pdf]

**An effective and concise device for detecting cold allodynia in mice**

Yonglan Ruan[1, 2, 3, 4 *], Leying Gu[1, 2, 3, 4 *], Jinjin Yan[1, 2, 3, 4], Jun Guo[1, 2, 3, 4], Xiao Geng[1, 2, 3, 4], Hao Shi[1, 2, 3, 4], Guang Yu[1, 2, 3, 4], Chan Zhu[1, 2, 3, 4], Yan Yang[1, 2, 3, 4], Yuan Zhou[1, 2, 3, 4], Changming Wang[1, 2, 3, 4 #], Zongxiang Tang[1, 2, 3, 4 #]

1. School of Medicine and Life Sciences, Nanjing University of Chinese Medicine, 138 Xianlin Rd, Nanjing 210023, Jiangsu, China
2. Key Laboratory of Chinese Medicine for Prevention and Treatment of neurological diseases, Nanjing University of Chinese Medicine, 138 Xianlin Rd, Nanjing 210023, Jiangsu, China
3. State Key Laboratory Cultivation Base for TCM Quality and Efficacy, Nanjing University of Chinese Medicine, Nanjing, 210023, China
4. Key Laboratory of Drug Target and Drug for Degenerative Disease of Jiangsu Province, Nanjing University of Chinese Medicine, Nanjing, 210023, China

*#Co-first authors:* Yonglan Ruan, Leying Gu

**Corresponding authors:*

Changming Wang

Nanjing University of Chinese Medicine

138 Xianlin Rd, Nanjing, JS 210023, China

Phone: +86-025-85811802

E-mail: bychangming@163.com

Zongxiang Tang

Nanjing University of Chinese Medicine

138 Xianlin Rd, Nanjing, JS 210023, China

Phone: +86-025-85811802

E-mail: zongxiangtang@njutcm.edu.cn

**Video 1. The video of the lifting behavior (cold allodynia) by the new device.**
